# Who says what to whom through what channel? Formative communication research on antibiotic resistance messaging for urgent care patients

**DOI:** 10.1017/ash.2024.429

**Published:** 2024-10-15

**Authors:** Rachel B. Wade, Monique M. Turner, Rana F. Hamdy, Youjin Jang, Ruth J. Heo, Cindy M. Liu

**Affiliations:** 1 Department of Communication, School of Arts and Sciences, The Ohio State University, Columbus, OH, USA; 2 Department of Communication, College of Communication Arts and Sciences, Michigan State University, East Lansing, MI, USA; 3 Children’s National Medical Center, Washington, DC, USA; 4 Communicating in Health Impact (CHI) Lab, University of North Carolina, Chapel Hill, NC, USA; 5 Antibiotic Resistance Action Center, Department of Environmental and Occupational Health, Milken Institute School of Public Health, George Washington University, Washington, DC, USA

## Abstract

**Objective:**

To explore the source, message, channel, and receiver effects on patient concern for antibiotic resistance, willingness to reduce antibiotic use, and expectations for an antibiotic prescription in a prepandemic sample.

**Methods:**

We used data reported from a national cross-sectional survey of adults who had visited an urgent care center within the last year. Data were collected from April 4 to April 9, 2017. The survey included an embedded experimental design to test changing effects before versus after message exposure.

**Participants:**

A national sample of adult participants (n = 610) who had used urgent care at least once in the past year were recruited through GfK’s KnowledgePanel^TM^. KnowledgePanel survey response rates are typically about 65%. Respondents ranged in age from 18 to 85 and were more likely to be female (377/610; 62%), White (408/610; 67%), and covered by private insurance (414/610; 68%).

**Results:**

Outcome variables were measured on 4-point scales 1–4 scale, and *t*-tests were conducted for measures that were collected pre and postmessaging. The majority of participants trusted their doctor and desired them as the source for information regarding antibiotic resistance, followed by field experts (eg, CDC). Direct messaging (eg, email) and targeted advertisements were least preferred.

**Conclusions:**

This study provides foundational data on patient communication preferences in terms of source, message content, and channel when receiving information on antibiotics and antibiotic resistance, as well as how these factors affect patient concern, willingness, and expectations. Follow-up work is needed to replicate these findings in a postpandemic sample.

## Introduction

Antibiotic resistance is a major global public health crisis. Annually, 2.8 million people in the U.S. are infected with an antibiotic-resistant strain, and approximately 35,000 people die due to resistance.^1^ The increase in antibiotic resistance and creation of antibiotic-resistant bacteria result from the overuse of antibiotics, inappropriate prescribing, extensive agricultural use, and a limited number of new antibiotics and alternatives. This leads to antibiotic-resistant bacteria that are resistant to even last-resort antibiotics, resulting in infections that become extremely difficult to treat and run the risk of spreading. Unfortunately, 1 in every 3 antibiotic prescriptions in outpatient settings were inappropriate for the presenting condition.^2^

Prior research has attempted to understand the patient’s role in the antibiotic prescribing process both for themselves and their children.^3,4^ Higher antibiotic expectations among patients have been shown to increase the likelihood of an antibiotic prescription.^5^ A patient’s previous experience with antibiotics can also result in self-diagnosis if they present with the same conditions. This results in increased patient expectations which, in turn, leads to patients accessing antibiotics through physician prescriptions, over-the-counter online, other countries, or a previous prescription. This issue is particularly prevalent among urgent care patients, who are much more likely to be inappropriately prescribed antibiotics than patients with similar afflictions seen in retail health clinics.^6^ Though one strategy is to address physician overprescribing practices, another tactic for understanding and mitigating overuse is patient-focused antibiotic stewardship.

Prior research has found patients are open to receiving targeted communication messages regarding antibiotic resistance.^7^ Moreover, providing information regarding antibiotic resistance has been shown to effectively mitigate inappropriate use.^8^ However, much of the work in this space has focused on making message content effective,^9,10^ with less being known about patients’ preferred methods for receiving these communications. To address this, we take a sender-message-channel-receiver (SMCR) approach. Though simplistic, this model first proffered by D.K. Berlo is foundational in communication studies research today.^9^

Communication scholars and practitioners believe it to be axiomatic that before an effective campaign can be launched, it is critical to understand preferences for who (source) says what (message) through what channel (modality) to whom (target audience) to achieve particular effects.^9^ Preferences for SMCR vary greatly across audiences and contexts; but campaign strategies cannot be developed in full without understanding who the audience trusts, the arguments they will find convincing, and the channels they prefer. Therefore, the variance of SMCR, and subsequent effects, was the focus of this formative research conducted with a national sample of urgent care patients. Urgent care patients were selected given that inappropriate prescription for respiratory conditions is highest in this patient population.^12^ Our objective was to explore each of these components to determine which elements resonate with patients to change their expectations. This study additionally explored the changing levels of antibiotic resistance concern, willingness to reduce use, and expectations for prescription before versus after message exposure.

## Methods

### Study design

A cross-sectional quantitative survey was conducted using a national sample of participants recruited through GfK’s KnowledgePanel^TM^, a probability-based online panel of adults that is recruited using address-based sampling. The survey was designed by the authors and Strategies 360, a Washington D.C.-based research firm contracted to implement the study. As part of their demographic profiling, GfK had collected data about urgent care use. This information was used in targeting potential survey participants, as well as in setting demographic quotas. Live telephone surveys were conducted between April 4 and 9, 2017.

The study received ethical approval from the George Washington University Human Subjects Review Board. Post hoc power analysis using G*Power (two-tail; *d* = .03; ɑ = 0.05; Power =.95) suggests a sample size of 580 to conduct means difference *t*-tests on independent groups. Considering Strategies 360’s predetermined margin of error for a survey of 610 is ±4.0% at the 95% confidence interval, the study was sufficiently powered. Though an exact response rate was not provided, the typical response rate for KnowledgePanel^TM^ is 65%.^13^

**Figure 1.**
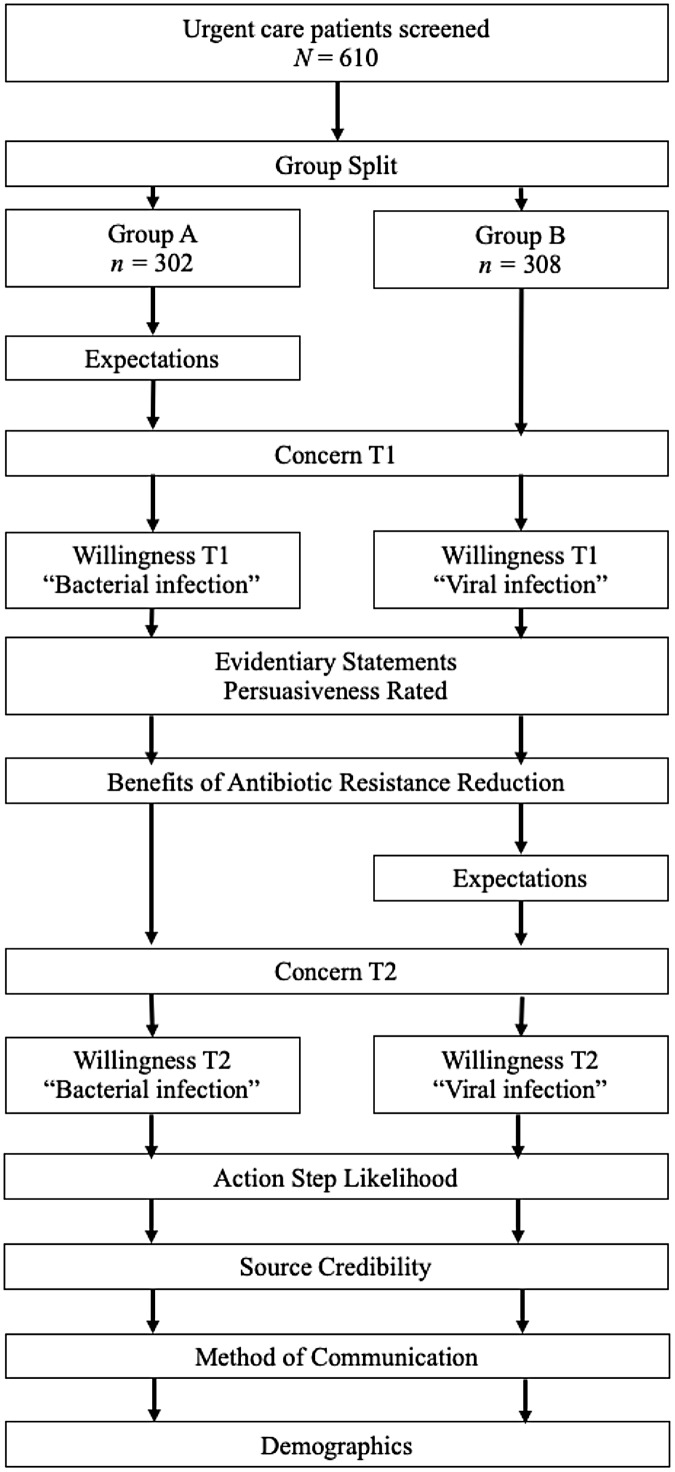
Flow chart of survey order and experimental design for group A and B.

All participants were asked to confirm they had been an urgent care customer within the last year, and about their health insurance and most frequently visited medical services (Table 1). The survey included one embedded, randomized, experimental component (Figure 1). This design allowed us to make some inferences about the persuasive potential of the messages. Half of the participants (Group A) were asked about their expectations regarding antibiotic prescription *before* viewing persuasive evidentiary messages. The other half (Group B) received these questions *after* viewing the messages. Moreover, those in Group A were asked about their willingness to reduce antibiotic use for “bacterial infections” and trust in “your doctor” and Group B participants were asked the same questions but about “viral infections” and “an urgent care doctor,” respectively (Table 2). For all other questions, Groups A and B were treated the same. In addition, all participants responded before and after viewing persuasive messages regarding their concerns and willingness to reduce use.

**Table 1.** Sample demographics

Variables	n (%)
**Age**	
18–24 years	66 (10.5)
25–34	153 (25)
35–44	124 (20.5)
45–54	91 (14.8)
55–64	96 (15.6)
65–74	68 (10.9)
75+	17 (2.7)
**Sex**	
Male	233 (38.2)
Female	377 (61.8)
**Race/Ethnicity**	
White, non-Hispanic	408 (66.8)
Black, non-Hispanic	75 (12.3)
Hispanic	91 (14.9)
2+ Races (non-Hispanic)	12 (2.0)
Other	24 (4.0)
**Education**	
Less than high school	54 (8.8)
High school	159 (26.1)
Some college	194 (31.9)
Bachelor’s degree or higher	202 (33.2)
**Housing Type**	
A one-family house detached from any other house	408 (66.9)
A one-family house attached to one or more houses	53 (8.7)
A building with 2 or more apartments	115 (18.8)
Other (eg, mobile home, boat, RV, van, etc.)	34 (5.6)
**Insurance Type**	
Private insurance	411 (67.4)
Medicare insurance	88 (14.5)
Medicaid insurance	61 (10.0)
Tricare insurance	14 (2.2)
Veterans’ administration	6 (1.0)
COBRA	3 (.6)
None	21 (3.4)
**Medical Setting Most Often Visited**	
Urgent care center	124 (20.3)
Primary care or family doctor	424 (69.4)
Hospital or emergency room	14 (2.4)
Other types of clinic (eg, community clinic)	23 (3.8)
Other	19 (3.2)
Not sure	6 (.9)

All *n* values are out of n = 610.

**Table 2.** Source trust ratings

		Percentage (%) (*n/n*)				
Reported Trust of:	*M* (*SD*)	Very much	Somewhat	A little	Not at all	Don’t know/Refuse
Your doctor^a^	1.56 (0.84)	59.4 (177/299)	31.4 (94/299)	5.0 (15/299)	2.5 (7/299)	1.7 (5/299)
An urgent care doctor^b^	1.95 (1.05)	40.0 (122/304)	38.0 (115/304)	14.3 (43/304)	2.8 (8/304)	5.0 (15/304)
Scientists and experts who study disease and infections	1.70 (0.95)	53.0 (319/603)	32.3 (195/603)	9.3 (56/603)	2.2 (13/603)	3.2 (19/603)
The Center for Disease Control and Prevention (CDC)	1.80 (1.08)	52.6 (317/603)	27.1 (164/603)	12.1 (73/603)	3.5 (21/603)	4.7 (28/603)
Medical professional organizations (eg, American Medical Association)	1.83 (1.00)	46.3 (279/604)	34.7 (210/604)	12.1 (73/604)	3.4 (20/604)	3.6 (22/604)
Nurses	1.92 (0.97)	38.2 (230/602)	41.5 (249/602)	13.9 (84/602)	3.1 (19/602)	3.3 (20/602)
The World Health Organization	2.15 (1.17)	35.4 (214/604)	34.7 (210/604)	16.1 (97/604)	7.2 (44/604)	6.5 (39/604)
Your friends or family	2.64 (0.99)	11.3 (68/603)	34.3 (207/603)	38.0 (229/603)	11.7 (71/603)	4.7 (28/603)

a Indicates question that only Group A answered.

b Indicates question that only Group B answered. All other questions were reported by both Group A and B. Likert scaled 1 “very much” to 4 “not at all.”

### Measures

The survey included 69 questions, including nine demographic variables, nine questions about urgent care perceptions, two questions capturing health literacy, and 49 questions about antibiotics.

#### Source

##### Credibility

Participants indicated how trustworthy they found message sources to be from 1 “very much” to 4 “not at all.” Examples include doctors, the World Health Organization (WHO), and friends/family (Table 2).

#### Message

##### Persuasiveness of evidentiary statements

Nine potentially persuasive arguments were presented halfway through the survey. Messages were intended to be diverse and spanned from biological to economic and social reasons. To gauge the persuasiveness of these evidentiary statements, participants were asked to mark how “convincing a reason” each statement was in helping to reduce antibiotic resistance on a scale from 1 “very convincing” to 4 “not convincing at all” (Table 3).

**Table 3.** Messages: ratings of how convincing each message was and ranking of benefits to help reduce antibiotic resistance

	Percentage (%) (*n/n*)					
Reported Convincingness of:	*M* (*SD*)	Very	Somewhat	A little	Not at all	Don’t know/Refuse
Taking antibiotics when you do not need them is really bad for you. It kills good bacteria which could make you even sicker.	2.11 (1.18)	38.5 (235/606)	30.5 (186/606)	18.0 (110/606)	5.7 (35/606)	6.7 (41/606)
Taking antibiotics when you do not need them can cause allergic reactions that could require going to the emergency room.	2.63 (1.27)	22.5 (137/606)	27.6 (168/606)	23.4 (143/606)	16.0 (97/606)	10.0 (61/606)
Taking antibiotics when you do not need them could make you up to 10 times more likely to get some potentially deadly infections.	2.11 (1.22)	41.0 (250/606)	26.9 (164/606)	19.2 (117/606)	4.4 (27/606)	7.9 (48/606)
Antibiotic resistance would make common medical procedures very hard or impossible, including surgery, childbirth, transplants, and cancer screenings.	2.10 (1.24)	41.4 (253/607)	28.7 (175/607)	15.1 (92/607)	6.5 (40/607)	7.8 (48/607)
Antibiotic-resistant bacteria could turn even a simple cut or scrape into a life-threatening or deadly illness.	2.12 (1.26)	42.6 (260/605)	24.8 (151/605)	16.4 (100/605)	7.8 (47/605)	7.6 (46/605)
Antibiotic resistance is a bigger problem for kids because they can only tolerate certain prescription medications, which puts kids at higher risk for long hospital stays or even death.	2.33 (1.29)	33.3 (203/606)	28.9 (176/606)	17.6 (107/606)	10.2 (62/606)	9.3 (57/606)
There is an easy, cheap solution to this problem. Science shows that if we’re smart about antibiotics and only take them when they are necessary and effective, many of the superbugs will lose their ability to resist antibiotics.	2.15 (1.18)	36.6 (223/606)	30.9 (189/606)	18.4 (112/606)	7.2 (44/606)	6.3 (38/606)
Antibiotic-resistant infections cost the U.S. healthcare system $26 billion a year, driving up healthcare costs for everyone.	2.38 (1.31)	31.2 (190/606)	30.1 (184/606)	19.0 (116/606)	7.8 (47/606)	11.4 (69/606)
Taking antibiotics when you don’t need them kills the good bacteria in your body which can cause problems like weight gain.	2.59 (1.27)	25.2 (154/607)	25.8 (157/607)	22.4 (137/607)	16.6 (101/607)	9.4 (57/607)

Likert scaled 1 “very convincing” to 4 “not convincing at all.”

##### Benefits of antibiotic resistance reduction

Participants were asked, “In your own words, what is the most important reason to help reduce antibiotic resistance?” Responses were recorded verbatim and coded to fit nine a priori thematic codes (Table 4).

**Table 4.** Preferences for channels and benefits of antibiotic resistance reduction

Method of Communication	n (%)
Your doctor or other healthcare professional	441 (72.3)
Healthcare websites online	214 (35.0)
Professional medical journals or medical magazines	181 (29.6)
A doctor you know personally, but is not your doctor	173 (28.4)
Google/search engines	133 (21.8)
Pamphlets or posters in your doctor’s office	121 (19.8)
National news outlets	95 (15.6)
Friends & family	79 (12.9)
Local news outlets	66 (10.8)
Social media (eg, Facebook, Twitter)	43 (7.1)
Online videos (eg, YouTube)	22 (3.6)
Email	15 (2.4)
Online advertising	10 (1.6)
Not sure	27 (4.5)

Benefit of Antibiotic Resistance Reduction	*n* (%)
To prevent superbugs	104 (25)
So antibiotics will work when we need them	100 (24)
To prevent routine illnesses from becoming threatening	79 (19)
Health (general)	29 (7)
Society health	29 (7)
To protect oneself/so your body can fight	25 (6)
Only use them when necessary	21 (5)
To reduce healthcare costs	8 (2)
Children/future generations	0 (0)
Other (not applicable)	41 (10)
Not sure	25 (6)

All *n* values for the preferred methods of communication are out of n = 610. Participants could choose up to 3 channel options. All the *n* values for the perceived benefits of antibiotic resistance reduction are out of n = 416 due to 194 participants opting not to give an open-ended response. Participant responses were recorded verbatim and coded into one of nine predetermined thematic codes.

#### Channel

##### Method of communication

Participants were asked, “Which 3 of the following ways would you most like to get information about antibiotic resistance?” then shown a list of 13 potential communication channels in a randomized order (Table 4).

#### Antibiotic resistance attitudes and behaviors

##### Concern

Concern about antibiotic resistance was measured before and after exposure to evidentiary statements from 1 “Very concerned” to 4 “Not concerned at all” but then reverse-coded such that greater values indicated more concern (Table 5).

**Table 5.** Evidentiary message effects of antibiotic expectation by symptoms, antibiotic resistance concern, and action step willingness

Symptom	*n*_*A*_ */n*_*b*_	Pre-message (Group A)	Post-message (Group B)	95% confidence interval of mean difference		*P*	*d*
		*M* (*SD*)	*M* (*SD*)	LL	UL		
Cough	279/289	2.07 (0.82)	1.69 (0.86)	–0.52	–0.24	<.001	–0.45
Fever	278/298	2.31 (0.90)	2.22 (1.05)	–0.25	0.07	.27	–0.09
Ear pain	283/289	2.77 (0.77)	2.67 (0.93)	–0.24	0.04	.17	–0.12
Sore throat	284/288	2.63 (0.76)	2.49 (0.93)	–0.28	–0.01	.04	–0.17
Stomach ache	285/283	1.64 (0.70)	1.53 (0.77)	–0.23	0.01	.07	–0.15
Deep cut/scrape	285/288	2.72 (0.89)	2.71 (0.99)	–0.17	0.13	.81	–0.02

Changes over time	*n*	Time 1	Time 2	95% Confidence interval of mean difference		*p*	*d*
		*M* (*SD*)	*M* (*SD*)	LL	UL		
Concern	547	2.91 (0.84)	3.23 (0.75)	–0.36	–0.25	<.001	–0.44
Willingness Group A	256	3.15 (0.66)	3.27 (0.67)	–0.19	–0.05	<.001	–0.22
Willingness Group B	260	3.41 (0.74)	3.52 (0.72)	–0.18	–0.03	.006	–0.17

Group A n = 302. Group B n = 308. Expectation for antibiotics by symptom was measured on a 4-point scale and reverse-coded so higher numbers indicate *greater* expectation. Concern and willingness were also measured on 4-point Likert scales and reverse-coded so higher values indicate *greater* concern/willingness.

##### Expectations

Six distinct symptoms^1^ (ie, ear pain, a deep cut/bad scrape, sore throat, fever, cough, stomachache) were presented to participants in random order. Participants were asked how likely they would be to expect antibiotics for each symptom from 1 “definitely expect” to 4 “definitely not expect” (reverse-coded; Table 5). Half of the sample reported expectations before exposure to evidentiary statements (Group A) and the other half reported after exposure (Group B).

##### Willingness

Willingness to help reduce antibiotic resistance was measured before and after exposure to persuasive messaging from 1 “Very willing” to 4 “Not willing at all” (reverse-coded; Table 5). Participants were asked, “To help reduce antibiotic resistance, how willing would you be to use antibiotics less often for [bacterial/viral] infections?” Half of the sample answered the bacterial question (Group A), the other half (Group B) answered the viral question.

##### Action steps

Participants indicated how likely they would be to take behavioral steps (eg, asking a provider if there are steps to take to feel better without antibiotics) after exposure to evidentiary statements. Responses ranged from 1 “very likely” to 4 “not likely at all.”

### Statistical analysis

All analyses were completed with SPSS 29. For measures that were collected pre- and postmessaging, a paired samples *t*-test was used to test for changes in response to messages. For measures that were collected across Groups A and B, an independent samples *t*-test was used to test for changes in response to messages. Our predetermined α level was .05.

## Results

### Sample results

Adults (n = 610) who had used urgent care at least once in the past year completed this survey (Table 1). Most respondents were 18–44 years old (341/610; 56%), and 53% (322/610) were married. Females comprised 62% (377/610) of the sample, 67% (408/610) were White non-Hispanic, and 26% (158/610) were parents to a 0- to 17-year-old child at the time of the study. Almost all were covered by health insurance (583/610; 95%) with most having private insurance (411/610; 67%).

### Survey results

#### Source

##### Credibility

Considering the trustworthiness of the message source (Table 2), around 60% (177/299) of the participants placed high trust in their doctor to inform them about the dangers and implications of antibiotic resistance. This was closely followed by 53% strongly trusting both the CDC (317/603) and experts and scientists who study infections (319/603). Only 40% (122/304) “strongly trusted” an urgent care doctor, 38% (230/602) a nurse, 35% (214/604) WHO, and 11% (68/603) their friends and family. Family and friends were shown to have the least amount of trust when concerning information about antibiotic resistance with 50% (300/603) trusting them “a little” or “not at all.”

#### Messages

##### Persuasiveness of evidentiary statements

The single most convincing message was “antibiotic-resistant bacteria could turn even a simple cut or scrape into a life-threatening or deadly illness” (260/605; 43%). Moreover, 70% of the respondents found the messages about threats to “common procedures,” (428/607) and antibiotics killing “good bacteria” (421/606) very or somewhat convincing (Table 3). The least convincing messages (ie, a little or not at all convincing) referenced the threat of an “allergic reaction” (240/606; 40%) or “weight gain” (238/607; 39%).

##### Benefits of antibiotic resistance reduction

One-fourth of the sample (104/416) found “to prevent superbugs” to be one of the most compelling motivators for reducing antibiotic resistance. Similarly, 24% (100/416) reported having antibiotics work when we need them was important, followed by the prevention of routine illnesses from becoming threatening (79/416; 19%). Other motivators were individually selected less than 10% of the time (Table 4).

#### Channel

##### Method of communication

Most participants (441/610; 72%) preferred to receive information on antibiotic resistance from their doctor or healthcare professional (Table 4). Other frequently selected channels were healthcare websites (214/610; 35%), professional medical journals (181/610; 30%), and a doctor they know personally but who is not *their* doctor (173/610; 28%). The least preferred methods were direct communication such as online advertising (10/610 2%), emailing (15/610; 2%), online videos (22/610; 4%), and social media (43/610; 7%). They also infrequently preferred local (66/610; 11%) or national news (95/610; 16%).

### Experimental results

#### Antibiotic resistance attitudes and behaviors

##### Expectations

Regarding patients’ expectations for receiving antibiotics for certain symptoms, results of an independent samples *t*-test (ie, differences between Groups A and B; Table 5) found that exposure to evidentiary statements decreased average expectations for cough with a mean difference of -0.38, 95% CI [–0.52, –0.24], *t*_*565.8*_ = -5.39, *P* <.001, Cohen’s *d* = -0.45. Expectations for sore throat also decreased, *M*_DIFF_ = –0.14, 95% CI [–0.28, –0.01], *t*_549.4_ = -2.02, *P* < .05, *d* = -0.17.

##### Concern

Exposure to evidentiary statements did increase concern about antibiotics. According to a paired samples *t*-test, this change in mean was significant, *M*_DIFF_ = 0.32, 95% CI [0.36, 0.25], *t*_*546*_ = 10.35, *P* < .001, *d* = 0.44 (Table 5).

##### Willingness

We conducted paired samples *t*-tests to test the change in participants’ willingness to reduce antibiotic use for bacterial and viral infections, detecting a 0.12 (95% CI = 0.19, 0.05; *P* < .001) and 0.11 (95% CI = 0.18, 0.03; *P* < .01) increase in mean willingness to engage in specific behaviors postmessaging, respectively (Table 5).

##### Action steps

Around 82% (491/602) of respondents stated that they were very/somewhat likely to not pressure their healthcare professional to prescribe antibiotics. However, more participants (516/602; 86%) were very/somewhat likely to ask their doctor for alternative steps to taking antibiotics when they were feeling ill. On the other hand, talking to a friend about the dangers of antibiotic resistance was the least popular action step, with only 67% (403/602) of the participants indicating that they were very/somewhat likely to do so (Table 6).

## Discussion

This article explores the effects of communication strategies surrounding antibiotic resistance. Results provide a rudimentary knowledge of patients’ preferences in sources, message content, channels, and their expectations related to antibiotic resistance. Understanding how these communication features resonate with patients may allow providers and practitioners to better communicate topics related to antibiotic resistance.

People not only trusted doctors as a source, but they also preferred to use them as a channel for information on antibiotic resistance. Around 60% (177/299) of participants placed high trust in their doctor to inform them about the danger and implications of antibiotic resistance, and 72% (441/610) indicated them as a preferred channel. This distinction is important, given that doctors both possess the relevant knowledge to convey (ie, information source) and are the conduit through which this information is interpersonally shared with patients (ie, channel). These findings indicate their ability to effectively play both roles.

Email and targeted advertisements were least preferred methods of communication, as most participants preferred to receive this information from a trusted medical source, namely their doctor or another healthcare provider. This suggests that, although widely available, direct messages may not be the most effective way to address patients regarding antibiotic resistance. Not only is it important for a trustworthy source (eg, doctor, field experts, CDC) to provide these messages, but they must also do so in an appropriate channel (eg, interpersonally, through a healthcare website, in a medical magazine).

Messages that focused on the *threat* of resistance (eg, antibiotics not working when needed; Table 3) were perceived as the most convincing. These findings are largely in line with prior research that has successfully implemented fear appeals to encourage proper antibiotic use.^10^ Importantly, these messages must be coupled with empowering content that maintains patients’ feelings of efficacy (ie, that they are capable of completing the proposed action) to be effective.^9^

This focus on threat coincided with perceived benefits of resistance reduction, such that participants found prevention of superbugs and rendering antibiotics effective, specifically for self, to be the most relevant benefits. The emphasis on the self is somewhat at odds with current recommendations to “emphasize that this is a universal issue.”^14^ Only 7% (29/416) of participants noted societal health as a benefit in antibiotic resistance reduction. Thus, we recommend perhaps the ”it affects everyone, including *you*” (our emphasis) is the critical element here.^14^

Postmessaging, individuals identified that they were very/somewhat likely to ask their doctor or healthcare professional if there were other steps they could take to feel better without taking antibiotics (516/602; 86%), as well as to promise never to pressure their healthcare provider to prescribe antibiotics (491/602; 82%). However, they were not as likely to talk to close friends and family about the dangers of antibiotic resistance, with 27% of respondents saying they were not likely to do this (Table 6). Given that family/friends are considered relatively undesirably methods of communication (Table 4), these messages appear effective for driving patients to their trusted source and preferred channel.

**Table 6.** Likelihood of taking particular behavioral action steps (postexposure to messaging only)

		Percentage (%) (*n/n*)				
Behavior	*M* (*SD*)	Very Likely	Somewhat Likely	Not Very Likely	Not Likely at All	Not Sure
Ask your doctor or healthcare professional if there are steps you can take to feel better without antibiotics.	1.74 (0.96)	49.1 (300/602)	35.5 (216/602)	8.6 (53/602)	1.9 (12/602)	3.5 (22/602)
Talk to your family and friends about the dangers of antibiotic resistance.	2.23 (1.12)	28.4 (173/602)	37.7 (230/602)	19.6 (119/602)	7.5 (46/602)	5.5 (34/602)
Promise to never pressure your doctor or healthcare professional to give you an antibiotic.	1.77 (1.11)	55.7 (340/602)	24.7 (151/602)	9.3 (57/602)	4.0 (24/602)	5.1 (31/602)

Items were measured on a 4-point Likert scale from 1 “very likely” to 4 “not likely at all.”

When measuring message effects, we saw a significant (*P <* .001) increase in concern about antibiotic resistance as well as an increase in willingness to reduce antibiotic use for both bacterial (Group A; *P* < .001) and viral (Group B; *P* < .01) infections. We also saw a decrease in participants’ prescription expectations for some symptoms. Expectation for cough (*P* < .001), and sore throat (*P* < .05) were significantly decreased after being exposed to the message.

### Limitations

Most notably, this survey was conducted in 2017 before the COVID-19 pandemic. Research has found that the pandemic impacted the U.S. public’s impression of the CDC as well as general knowledge about the effectiveness of antibiotics against viral infections.^15^ Future efforts will need to be taken to replicate these findings in newer samples with additional questions about antibiotic use to understand how the pandemic and drug usage may have influenced the relationships found here.

In addition, this study does not examine SMCR preferences by target audience variables.^16^ For example, it could be the case that more educated audiences have distinct preferences from those with significantly less education. Moreover, communication research has made clear that targeting by demographics is the least satisfactory form of targeting.^17^ Rather, message designers (eg, marketing firms) prefer to target by psychographics such as lifestyle or culture.^17^ This was beyond the scope of this study but should be carefully considered in future research on antibiotic resistance messaging.

Finally, though results showed that individuals were more concerned with antibiotic resistance and more willing to reduce antibiotic use after being presented with persuasive messaging, we presented nine messages to participants at once and thus do not know which standalone messages are necessarily the most effective. A conjoint analysis that manipulates SMCR elements to compare is a welcome future direction.

## Conclusion

These results offer healthcare providers as a useful source and channel for antibiotic resistance information sharing. Furthermore, practitioners may find these results useful in designing a communication campaign message with appropriate messages for patients. We believe that even a basic understanding may allow providers and practitioners to better intervene to reduce antibiotic resistance, whether it be used in clinical care, teaching, or designing health communication campaigns.

## Supplementary material

For supplementary material accompanying this paper visit http://doi.org/10.1017/ash.2024.429. click here to view supplementary material

## Supporting information

Wade et al. supplementary material Wade et al. supplementary material

## Footnotes

1
In this study, our position was that patients should not expect antibiotics until a provider provides a diagnosis. We recognized that some symptoms were more likely to correlate with the need for an antibiotic--and therefore presented all of the expectation data (versus only an aggregate).

## Research transparency and reproducibility

Deidentified data available upon request.
