# Supplementary material for: Who says what to whom through what channel? Formative communication research on antibiotic resistance messaging for urgent care patients
Source: Antimicrob Steward Healthc Epidemiol. 2024 Oct 15;4(1):e177. doi: 10.1017/ash.2024.429 (PMC11474784; doi:10.1017/ash.2024.429)
Supplement: Supplementary file 1 [file S2732494X24004297sup001.pdf]

Appendix A: Survey Instrument

A National Survey of Urgent Care Patients

April 4-9, 2017 | *N* = 610; Margin of Error is ±4.0%

1. Overall, do you have an easy or hard time staying healthy?
Very easy
Somewhat easy
Somewhat hard
Very hard
Not sure

---

2. Do you currently have health insurance?
Yes, private insurance
Yes, Medicare
Yes, Medicaid
Yes, Tricare
Yes, Veterans Administration
Yes, COBRA
No
Not sure

(RANDOMIZE RESPONSE ORDER)
3. Which of these medical settings do you visit most often?
Urgent care center
Primary care or family doctor
Hospital or emergency room
Other type of clinic, such as a community clinic
Other (specify
Not sure

---

4. In the last year, how many times have you visited an urgent care center?
0
1
2-5
6-10
More than 10
Not sure

---

5. In general, how satisfied are you with the care and treatment you've gotten at urgent care centers?
Very satisfied
Somewhat satisfied
Mixed
Somewhat dissatisfied
Very dissatisfied
Not sure

(RANDOMIZE RESPONSE ORDER)
6. For which of the following reasons do you visit an urgent care center? Please mark all that apply.
My primary care or family doctor was not available the same day
My primary care or family doctor was not available within 48 hours

They offer after-hours access
I don’t have a primary care or family doctor and this was a good option
Other **(SPECIFY)**
Not sure

---

7. Do you have a primary care doctor? This is a doctor you see for checkups and common health problems.
Yes, and I’ve seen them at least once in the past 3 years
Yes, but I haven’t seen my primary care doctor in the past 3 years
No, but I have had one who I’ve seen within the last 3 years
No, I haven’t had a primary care doctor who I’ve seen in the last 3 years
Not sure

---

8. Would you trust advice from an urgent care doctor more, less, or the same as advice from your primary care doctor or family doctor?
More
The same
Less
Not sure

---

9. Imagine you need to see a doctor quickly, but you can’t get an appointment with your primary care doctor fast enough. Regardless of the type
of illness or injury you have, how confident are you in making a decision about whether you need to go to an urgent care center, a hospital
emergency room, or somewhere else?

Very confident
Somewhat confident
Not too confident
Not confident at all
Not sure

---

10. This information is on the back of a container of a pint of ice cream. Please use the label to answer these 6 questions.

NUMBER OF CORRECT RESPONSES
0-1 (high likelihood of limited literacy)
2-3 (possibility of limited literacy)
4-6 (adequate literacy)

---

11. How confident are you filling out medical forms by yourself?
Extremely
Quite a bit
Somewhat
A little bit
Not at all
Not sure

---

12. Please mark if you think the following statement is true or false, and how certain you are about your answer.
Antibiotics work for both bacterial and viral infections.
True, I am certain
True, my best guess
False, my best guess
False, I am certain

Not sure

(SPLIT A, ½ SAMPLE)

Below are some symptoms that some people might visit an urgent care center for. For each symptom, please mark how likely you would be to expect antibiotics.

Definitely expect
Probably expect
Probably not expect
Definitely not expect
Not sure

RANDOM ROTATE ALL:
13. Ear pain
14. A deep cut or bad scrape
15. Sore throat
16. Fever
17. Cough
18. Stomach ache

---

19. On average, how many times a year do you take antibiotics?
0
1
2-5
6-10
More than 10
Not sure

---

20. Have you ever asked a doctor or health care professional for antibiotics?
Yes
No, but I would
No, and I would not
Not sure

---

21. **(ONLY ASK OF PARENTS WITH CHILDREN UNDER AGE 18)**
Have you ever asked a doctor or health care professional for antibiotics for your children?
Yes
No, but I would
No, and I would not
Not sure

---

22. How much would you say you know about antibiotic resistance? You are not expected to be an expert on this topic or have all the answers.
A lot
A little
Nothing at all
Not sure

---

23. Based on what you know or have heard, how concerned are you about antibiotic resistance?
Very concerned
Somewhat concerned

Not very concerned
Not concerned at all
Not sure

24.
(SPLIT A) To help reduce antibiotic resistance, how willing would you be to use antibiotics less often for **bacterial infections?**
(SPLIT B) To help reduce antibiotic resistance, how willing would you be to use antibiotics less often for **viral infections?**

Very willing
Somewhat willing
Not very willing
Not willing at all
Not sure

25. Just so that everyone taking this survey has the same information, please read the following statement. Antibiotic resistance means that bacteria can learn to avoid the effects of an antibiotic. Bacteria that can resist antibiotics are often called superbugs, and they are harder to kill. That means that antibiotics that cure or prevent infection from bacteria don't work as well. Antibiotic resistance is caused by the use and overuse of antibiotics.

For each statement below, please mark how convincing a reason it is to help reduce antibiotic resistance.

Very convincing
Somewhat convincing
A little convincing
Not convincing at all
Not sure

RANDOM ROTATE ALL:

26. **[COMMON PROCEDURES]** Antibiotic resistance would make common medical procedures very hard or impossible, including surgery, childbirth, transplants, and cancer screenings.

27. **[KILLS GOOD BACTERIA]** Taking antibiotics when you don't need them is really bad for you. It kills good bacteria, which could make you get even sicker.

28. **[SCRAPE OR CUT]** Antibiotic-resistant bacteria could turn even a simple cut or scrape into a life-threatening or deadly illness.

29. **[10X MORE LIKELY]** Taking antibiotics when you don't need them could make you up to 10 times more likely to get some potentially deadly infections.

30. **[ASPIRATIONAL]** There is an easy, cheap solution to this problem. Science shows that if we're smart about antibiotics and only take them when they are necessary and effective, many of the superbugs will lose their ability to resist antibiotics.

31. **[KIDS]** Antibiotic resistance is a bigger problem for kids because they can only tolerate certain prescription medications, which puts kids at higher risk for long hospital stays or even death.

32. **[COSTS]** Antibiotic-resistant infections cost the U.S. health care system $26 billion a year, driving up health care costs for everyone.

33. **[WEIGHT]** Taking antibiotics when you don't need them kills the good bacteria in your body which can cause problems like weight gain.

34. **[ALLERGIC REACTION]** Taking antibiotics when you don't need them can cause allergic reactions that could require going to the emergency room.

---

35. In your own words, what is the most important reason to help reduce antibiotic resistance? **[RECORD VERBATIM RESPONSE]***

To prevent superbugs
So antibiotics will work when we need them
To prevent routine illnesses from becoming threatening
Health (general)
Society health
To protect oneself/so your body can fight
Only use them when necessary
To reduce health care costs
Children/future generations
Other
Not sure
**N* = 416

---

36. Please tell me which of the following statements concerns you more.

Antibiotic resistance means that antibiotics will be less effective for me and may not work when I really need them.

OR

Antibiotic resistance means that we're creating superbugs that are harder to kill and could infect anyone.

(SPLIT B, ½ SAMPLE)
Below are some symptoms that some people might visit an urgent care center for. For each symptom, please mark how likely you would be to expect antibiotics.

Definitely expect
Probably expect
Probably not expect
Definitely not expect
Not sure

RANDOM ROTATE ALL:
37. Ear pain

38. A deep cut or bad scrape
39. Sore throat
40. Fever
41. Cough
42. Stomach ache

---

43. Now that you have more information, thinking again, how concerned are you about antibiotic resistance?

Very concerned
Somewhat concerned
Not very concerned
Not concerned at all
Not sure

44.
(SPLIT A) Thinking again, to help reduce antibiotic resistance, how willing would you be to use antibiotics less often for bacterial infections?
(SPLIT B) Thinking again, to help reduce antibiotic resistance, how willing would you be to use antibiotics less often for viral infections?

Very willing
Somewhat willing
Not very willing
Not willing at all
Not sure

Below are some ways that urgent care centers might help reduce antibiotic resistance. For each one, please imagine yourself as a patient in an urgent care center. Please mark how satisfied you would be if this situation happened to you.

Very satisfied
Somewhat satisfied
Not very satisfied
Not satisfied at all
Not sure

RANDOM ROTATE ALL:
45. During your visit to an urgent care center, the doctor does a test that can tell right away if antibiotics would help treat your illness.

46. During your visit to an urgent care center, the doctor explains to you that antibiotics won't be an effective treatment for your illness. However, they discuss your illness with you in-depth and come up with a plan to help you feel better without antibiotics.

47. **(SPLIT B)** During your visit to an urgent care center, the doctor explains that they don't know if your infection is viral or bacterial, so they can't know if antibiotics will help. But they say taking antibiotics if you don't need them could add to the problem of antibiotic resistance and make them less likely to work when you need them. The doctor offers to give you a prescription for

antibiotics, but dates it 2-3 days in the future to see if you get better without antibiotics.

48. **(SPLIT A)** During your visit to an urgent care center, the doctor explains that they don't know if your infection is viral or bacterial, so they can't know if antibiotics will help. But they say taking antibiotics if you don't need them could add to the problem of antibiotic resistance and make them less likely to work when you need them. The doctor offers to give you a prescription for antibiotics, but asks you to wait 2-3 days to see if you get better before you fill the prescription.

Below are some actions people could take to help reduce antibiotic resistance. For each, please mark how likely you would be to take that action.

Very likely
Somewhat likely
Not very likely
Not likely at all
Not sure

RANDOM ROTATE ALL:
49. Ask your doctor or health care professional if there are steps you can take to feel better without antibiotics.
50. Promise to never pressure your doctor or health care professional to give you an antibiotic.
51. Talk to your family and friends about the dangers of antibiotic resistance.

Below are some different people and organizations who may be involved in the issue of antibiotic resistance. For each, please mark how much you trust
this person or organization on this issue.

Very much
Somewhat
A little
Not at all
DK/REF

RANDOM ROTATE ALL:
52. **(SPLIT A)** Your doctor
53. Scientists and experts who study disease and infections
54. The Center for Disease Control and Prevention, or CDC
55. Medical professional organizations such as the American Medical Association
56. **(SPLIT B)** An urgent care doctor
57. Nurses
58. The World Health Organization
59. Your friends or family

---

60. Which 3 of the following ways would you most like to get information about antibiotic resistance? **(ACCEPT UP TO THREE RESPONSES)**
(RANDOMIZE LIST)

Your doctor or other health care professional
Health care websites online
Professional medical journals or medical magazines
A doctor you know personally, but is not your doctor
Google or other search engines
Pamphlets or posters in your doctor's office
National news outlets, like ABC News, FOX, or the New York Times
Your friends and family
Local news outlets, like your local TV station or newspapers
Social media, including Facebook and Twitter
Online videos, like YouTube
Online advertising
Email
Other **(specify)**
Not sure
Refused

DEMOGRAPHICS

61. Gender
Male
Female

62. Age
18-24
25-34
35-49
50-64
65+

63. Educational attainment
Less than high school
High school
Some college
Bachelor's degree or higher

64. Race/ethnicity
White, non-Hispanic
Black, non-Hispanic
Hispanic
Other (non-Hispanic)
2+ races (non-Hispanic)

65. Marital status
Married
Widowed
Divorced
Separated
Never married
Living with partner

66. Parental status
Parent of a 0-17 year old
Not a parent of a 0-17 year old

67. Region
Northeast
Midwest
South
West

68. Own/rent home
Own
Rent
Occupy without payment of cash rent

69. As of today, do you consider yourself a Republican, a Democrat, or an independent?
Strong Democrat
Not strong Democrat
Independent, lean Democratic
Independent
Independent, lean Republican
Not strong Republican
Strong Republican
Other
DK/NA/REFUSED
